# Overlapping holoprosencephaly‐polydactyl syndrome and asphyxiating thoracic dystrophy, an incidental finding in late prenatal ultrasound: A rare case report

**DOI:** 10.1002/ccr3.3836

**Published:** 2021-01-24

**Authors:** Senai Goitom Sereke, Anthony Oriekot, Felix Bongomin

**Affiliations:** ^1^ Department of Radiology and Radiotherapy School of Medicine Makerere University College of Health Sciences Kampala Uganda; ^2^ Department of Medicine School of Medicine Makerere University College of Health Sciences Kampala Uganda; ^3^ Department of Medical Microbiology and immunology Faculty of Medicine Gulu University Gulu Uganda

**Keywords:** alobar holoprosencephaly, Jeune syndrome, polydactyl, short ribs

## Abstract

Holoprosencephaly‐polydactyly syndrome and asphyxiating thoracic dystrophy rarely overlap but if they do, they have poorer prognosis. Early prenatal detection of multiple congenital anomalies plays a crucial role in the management of pregnancy.

## Graphical abstract

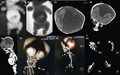

## 1 INTRODUCTION

An obstetric ultrasound of a multi‐gravid mother at 37‐week of gestation showed a female fetus with alobar holoprosencephaly, polydactyly, short ribs, narrow chest, and short upper and lower extremity bones, consistent with holoprosencephaly‐polydactyly syndrome and asphyxiating thoracic dystrophy overlap. Apgar score was 0 in the first and fifth minute.

Holoprosencephaly is a disorder resulting from failure of septation, cleavage, or differentiation of the midline forebrain structures at various levels or to various degrees. Defects in development of the midfacial region frequently coexist.^1^ To date, there are four variants of holoprosencephaly according to the degrees of failed differentiation: lobar, semilobar, alobar, and middle interhemispheric variant (syntelencephaly).^2^ The various holoprosencephalic abnormalities of the face include cyclopia (single eye or partially divided eye), proboscis (elongated nose), ethmocephalus (narrow‐set eyes with an absent nose and abnormal smallness of one or both eyes), cebocephaly (two separate eyes set close together, and a small, flat nose with a single nostril), premaxilla agenesis (least severe form of cebocephaly in the spectrum of facial anomalies is the median cleft lip), median cleft palate/lip, and other less‐severe facial dysmorphism.^1^, ^2^, ^3^

Holoprosencephaly–polydactyly syndrome (HPS), also known as pseudotrisomy 13, is one of the less understood congenital syndromes, and the criteria of diagnosis remains controversial. There is some significant overlap of HPS with other disorders such as hydrolethalus syndrome (exclusively from Finland), trisomy 13, which may lead to difficulties in establishing a diagnosis.^4^, ^5^

Asphyxiating thoracic dystrophy (ATD), also known as Jeune syndrome, was first described in 1955 and is a rare autosomal recessive skeletal dysplasia with multi‐organ involvement. It is characterized by a small, narrow chest and variable limb shortness with a considerable neonatal mortality as a result of respiratory distress. Other complication of the kidney, liver, pancreas, and the eye may occur later in life, if the fetus grows to term.^6^

Prenatal ultrasound is an important component of the antenatal care (ANC) package, which can be performed throughout gestational age to check the well‐being as well as early screening for fetal anomalies.^7^ Herein, we describe some incidental findings of a rare co‐occurrence of HPS and Jeune syndrome diagnosed prenatally through routine obstetric ultrasonography.

## 2 CASE PRESENTATION

In mid‐2019, we received a 31‐year‐old gravida 3, Para 1 + 1 (abortion) mother who came for a third trimester routine ANC follow‐up in Kawempe National Referral Hospital (KNRH), Kampala, Uganda. She was at her 37 weeks by last menstrual period. She received all routine ANC medications as per the Ugandan national guidelines. The pregnancy was uneventful. She already had a normal male child who was 3 years old. There was a first trimester abortion (at two months) before this current pregnancy. In her current pregnancy, she came once at 24 weeks for her booking ANC and missed her obstetric ultrasound scan. Her blood pressure was consistently in the range of 95/70‐110/75 mm Hg. She added 11 kg of weight during the pregnancy (from the 3rd month of the pregnancy to the time of presentation). Both parents were of Ugandan decent and in a non‐consanguineous marriage.

**FIGURE 1:**
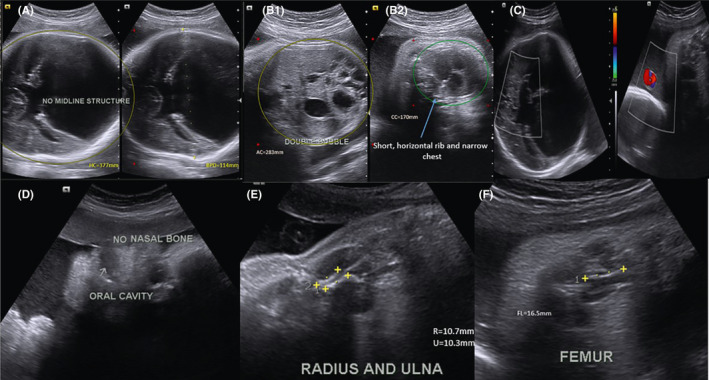
(A, B (1,2), C, D, E and F) – prenatal ultrasound. A, Demonstrates absence of midline structures and single ventricle, head circumference (377 mm) and biparietal diameter (114 mm) below > 95th percentile for gestational age. B1, Demonstrates abdominal circumference (283 mm) which was between 10th and 50th percentile, double bubble sign, B2, Narrow chest (Chest circumference = 170 mm), short and horizontal ribs which was <5th percentile. C, Color Doppler demonstrates no flow in the remained brain parenchyma and three‐vessel cord of umbilicus. D, Demonstrates absence of nasal bone. E, Demonstrates short radius (10.7 mm) and ulna (10.3 mm) which was <5th percentile. F, Demonstrates very short femur (FL = 16.5 mm) which was <5th percentile

On ultrasound examination, the fetus was in longitudinal lie and breech presentation. A two‐dimensional (2D) ultrasound scan was done on the fetus. The biparietal diameter (BPD) was 114 mm and head circumference was 377 mm (which was above 95th percentile for gestational age). There was absence of brain midline structures with fused thalami. The nasal bone was absent. The neck was short. The ribs were short with narrow chest (Chest circumference = 170 mm, which was below 5th percentile for gestational age) and four‐chambered heart with both right and left ventricular outlets were normal. The diaphragm was present. Double bubble sign, demonstrating a dilated stomach and duodenum was demonstrated in the abdomen (AC = 283 mm, between 10th and 50th percentile for gestational age). Both kidneys and urinary bladder were seen and normal. The femur (FL = 16.5 mm), tibia, fibula, humerus, radius (10.7 mm), and ulna (10.3 mm) were short (below 5th percentile for gestational age). There was three‐vessel umbilical cord. The amniotic fluid index (AFI) was 29 cm. The placenta was single lobed and was implanted anteriorly (Figure 1A‐F).

A sonographic impression of alobar holoprosencephaly, micromelia, duodenal atresia and short ribs, and narrow chest in breech presentation was made. The parents were informed about the abnormalities and the prognosis. They denied the occurrence of similar condition and other abnormalities from both paternal and maternal relations. The obstetrician believed that cesarean delivery was the only option as destructive delivery (given the unfavorable prognosis of overlapping syndrome of the fetus) was not plausible due to the breech presentation of the fetus. The baby was alive until the last minute of cesarean section.

**FIGURE 2:**
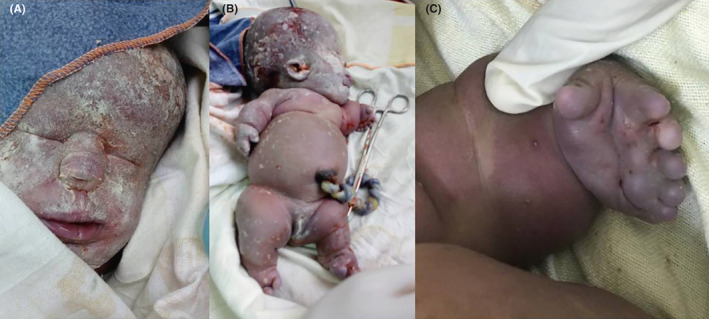
(A, B and C) post‐delivery images of the neonate. A phenotypically female neonate with big head, flat nose, low set ears, small chest, invariably short limbs,and polydactyly of four limbs

A 3.5 kg, phenotypically female baby with Apgar score of zero in the first and fifth minute was delivered through an elective cesarean section. The fetal demise was intrapartum, and attempt was made to resuscitate the neonate but failed. The neonate had six digits and seven digits on the left and right hands, respectively. The right and the left feet had five and four abnormally big toes, respectively. The head circumference was 45 cm and the sutures were widened. The nasal bridge was flat, low set ears, but no cleft lip. The chest circumference was 23 cm (less than the 5th percentile). Both upper and lower extremities were abnormally short (Figure 2A‐C). Informed consent was taken from parents for radiological postmortem evaluation, but, denied consent for an autopsy.

**FIGURE 3:**
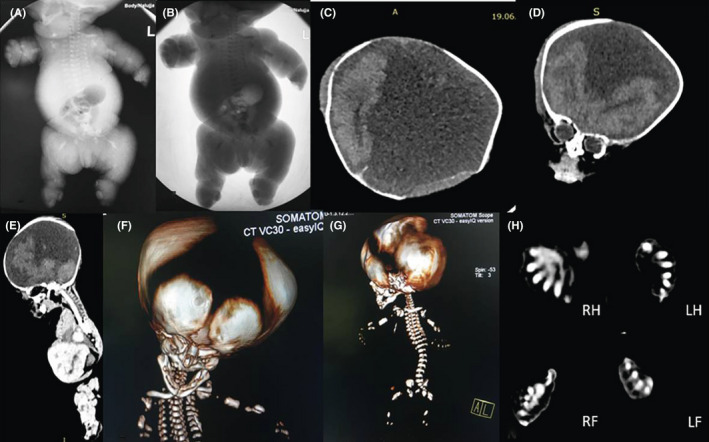
(A and B) postnatal radiography study. (C, D, and E) CT scan with brain window. (F and G) 3D CT bone reformat. (H) CT scan in bone window. (A and B) demonstrate short and horizontal oriented ribs, bilateral short humerus, and femur. (C, D and E) demonstrate absent midline structures with frontal lobes and occipital lobe in place. F) Demonstrates widely open cranial sutures. (G) Demonstrates short and horizontally oriented ribs. (H) demonstrates postaxial polydactyly of the Right hand (RH), Left hand (LH), Right foot (RF), and Left foot (LF)

The postnatal radiography study demonstrated short ribs, short long bones and double bubble sign in the abdomen (Figure 3A,B).

The whole‐body CT scan demonstrated widely open cranial sutures, absent midline structures, postaxial polydactyly of the hands and feet, short and horizontal oriented ribs and narrow chest; and variably short long bones. The phenotypically big toes (5 in the right and 4 in the left feet) showed overlapping phalanges (Figure 3C‐H).

## 3 DISCUSSION

Holoprosencephaly is the most common congenital malformation of the forebrain, occurring 1 in 20 000 live births.^8^ As observed in the present case, the intrauterine fetal death is common among infants with HPS.^4^ Although the present was phenotypically female, literature suggests that HPS predominantly affect the male sex.^4^ Despite the fact that cardiac and genitourinary abnormalities often accompany HPS in a majority of cases,^4^ this was not observed in the present report. To the best of the authors’ knowledge, this is the first case of HPS to be published in Uganda and the first case of HPS‐ATD overlap to be reported in the literature.

Holoprosencephaly–polydactyly syndrome are heterogeneous group of congenital malformations whose phenotype is consistent with that of trisomy 13 in the context of a normal karyotype‐ hence often referred to as pseudotrisomy 13.^9^ The following diagnostic criteria have been proposed: (a) a combination of holoprosencephaly and postaxial polydactyly with or without other characteristics; or (b) a combination of holoprosencephaly with other characteristics but without polydactyly; or (c) a combination of postaxial polydactyly, brain defects (microcephaly, hydrocephaly, agenesis of corpus callosum), and other characteristics.^10^ The neurologic outcome and mortality risk of holoprosencephaly is dependent on the severity of the disease, in which alobar has the worst prognosis. Fetuses with isolated alobar holoprosencephaly do not survive beyond early infancy period.^11^

The definitive diagnosis of HPS remains a challenge. Initially, polydactyly and normal chromosomal analysis served as the diagnostic criteria of HPS. However, some studies showed that not all cases of HPS have normal chromosomes. This indicated that even in the presence or absence of normal chromosome the diagnosis of HPS is clinical.^4^ The presented case was sporadic, and the phenotypic and the prenatal ultrasound features were highly suggestive of pseudotrisomy 13. As much as genome wide microdeletion or duplication analysis is ideal for the detection of aneuploidy, it was not done for it was not available in our setting.

Trisomy 13 shows a significant difference from HSP when a detailed phenotypic analysis is performed. In trisomy 13, cystic dysplasia or embryonal lobulation of the kidneys and hydronephrosis occur in 80% cases.^10^, ^12^ Grote syndrome has unique combination of holoprosencephaly, tetramelic octodactyly, heart defect, bilateral tibial agenesis, and multivisceral malformations.^13^ The baby had no cystic dysplasia and neither did it had hydronephrosis nor tetramelic octodactyly.

Asphyxiating thoracic dystrophy is a rare autosomal recessive skeletal dysplasia of unknown etiology with multi‐organ involvement. Diagnosis is solely based on clinical and radiographic findings. It is clinically characterized by a small, narrow chest and variable limb shortness. Postaxial polydactyly of both hands and/or feet is described as associated congenital abnormalities in 20% case.^14^ Radiographically it is typical characterized by a narrow, bell‐shaped thorax with short, horizontally oriented ribs and irregular costochondral junctions, elevated clavicles, short iliac bones with a typical trident appearance of the acetabula, relatively short and wide long bones of the extremities, and hypoplastic phalanges of both hands and feet with cone shaped epiphyses. In isolated syndromes, the small and narrow thorax often is associated with respiratory distress and recurrent respiratory infections in the postnatal and infancy period respectively.^15^, ^16^, ^17^ The present case had small, narrow chest, variable limb shortness, and postaxial polydactyly of both hands on clinical examination. Radiological findings showed short, irregular and horizontal oriented ribs, narrow and bell‐shaped thorax, and short iliac bones. The clinical and radiographic features suggested the presence of an overlapping syndrome other than an isolated HPS in the neonate.

Polymorphisms in several genes including IFT80, DYNC2H1, WDR19, IFT140, and TTC21B have been identified in some families with ATD.^6^ ATD and Ellis–van Creveld syndrome are clinically and radiographically similar disorders characterized by skeletal dysplasia. The most important distinguishing feature is, thoracic involvement is less pronounced in Ellis–van Creveld syndrome. Hence it has better prognosis than ATD.^18^, ^19^, ^20^ In the present case, the chest was profoundly affected.

Visualization of a fluid‐filled double bubble on prenatal ultrasound scan is associated with duodenal obstruction secondary to intrinsic or extrinsic cause. It can be associated with VACTERL (vertebral, anorectal, tracheoesophageal, renal, limb), chromosomal anomalies like trisomy 21 or it can be an isolated entity.^21^, ^22^ The presence of duodenal atresia was diagnosed on the prenatal late ultrasound and was confirmed by postmortem baby‐gram and was found in association with HPS and Jeune syndrome. It hasn't been described in connection with either of these syndromes in the literature.

## 4 CONCLUSION

Holoprosencephaly–polydactyly syndrome and asphyxiating thoracic dystrophy rarely overlap but if they do, they have poorer prognosis. Early prenatal detection of multiple congenital anomalies by routine ultrasound screening in the 18‐22 weeks gestation, plays a big role in the early management of pregnancy, delivery and postnatal complication to the fetus and the mother.

## CONFLICT OF INTEREST

None declare.

## AUTHORS’ CONTRIBUTION

All authors made substantial contributions to conception and design, acquisition of data, or analysis and interpretation of data; took part in drafting the article or revising it critically for important intellectual content; agreed to submit to the current journal; gave final approval of the version to be published; and agree to be accountable for all aspects of the work.

## Data Availability

The information used and/or analyzed during this case report is available from the corresponding author on reasonable request.
